# Bradykinin/B_2_ receptor activation regulates renin in M‐1 cells via protein kinase C and nitric oxide

**DOI:** 10.14814/phy2.13211

**Published:** 2017-04-03

**Authors:** Lucienne S. Lara, Camille R. T. Bourgeois, Samir S. El‐Dahr, Minolfa C. Prieto

**Affiliations:** ^1^ Instituto de Ciências BiomédicasUniversidade Federal do Rio de JaneiroRio de JaneiroBrazil; ^2^ Department of PhysiologyTulane University School of MedicineNew OrleansLouisiana; ^3^ Tulane Hypertension and Renal Center of ExcellenceTulane UniversityNew OrleansLouisiana; ^4^ Department of PediatricsSection of Pediatric NephrologyTulane University Health Sciences CenterNew OrleansLouisiana

**Keywords:** cGMP, distal tubular renin, gene expression, prorenin, protein kinase A

## Abstract

In the collecting duct (CD), the interactions of renin angiotensin system (RAS) and kallikrein‐kinin system (KKS) modulate Na^+^ reabsorption, volume homeostasis, and blood pressure. In this study, we used a mouse kidney cortical CD cell line (M‐1 cells) to test the hypothesis that in the CD, the activation of bradykinin B_2_ receptor (B_2_R) increases renin synthesis and release. Physiological concentrations of bradykinin (BK) treatment of M‐1 cells increased renin mRNA and prorenin and renin protein contents in a dose‐dependent manner and increased threefold renin content in the cell culture media. These effects were mediated by protein kinase C (PKC) independently of protein kinase A (PKA) because B_2_R antagonism with Icatibant and PKC inhibition with calphostin C, prevented these responses, but PKA inhibition with H89 did not modify the effects elicited by the B_2_R activation. BK‐dependent stimulation of renin gene expression in CD cells also involved nitric oxide (NO) pathway because increased cGMP levels and inhibition of NO synthase with L‐NAME prevented it. Complementary renin immunohistochemical studies performed in kidneys from mice with conventional B_2_R knockout and conditional B_2_R knockout in the CD, showed marked decreased renin immunoreactivity in CD, regardless of the renin presence in juxtaglomerular cells in the knockout mice. These results indicate that the activation of B_2_R increases renin synthesis and release by the CD cells through PKC stimulation and NO release, which support further the interactions between the RAS and KKS.

## Introduction

Renin release is the rate‐limiting step of the renin angiotensin system (RAS) cascade (Schweda et al. 2007). In response to reduction in extracellular fluid volume and blood pressure, this aspartyl protease is released by the granular cells from the juxtaglomerular apparatus (JGA) localized in the afferent arterioles (Rosa et al. 2016). For more than two decades, it has been known about the presence of renin transcripts (pre‐prorenin) and renin protein in the renal tubular segments with pronounced expression in the principal cells of the collecting ducts (CD) (Taugner et al. 1982; Rohrwasser et al. 1999; Prieto‐Carrasquero et al. 2004). This renin is named “CD renin”. In contrast to JGA renin, angiotensin (Ang) II feeds‐forward CD renin, whereas it inhibits JGA renin (Prieto et al. 2013). Angiotensin II‐dependent stimulation of CD renin is mediated by AT1 receptor (AT_1_R) activation independent of blood pressure (Mamenko et al. 2013) through a mechanism mediated by protein kinase C (PKC) and cAMP (Gonzalez et al. 2011b, 2015). Collecting duct renin is primarily produced during pathological conditions and serves as a source to increase de novo generation of intratubular angiotensin (Ang) II (Gonzalez and Prieto 2015a,b). This is functionally relevant since Ang II increases Na^+^ reabsorption in the CD via AT1R‐dependent stimulation of epithelial Na^+^ channel (ENaC) (Mamenko et al. 2013) and contributes to kidney damage (Cuevas et al. 2015). Indeed, even small adjustments in the amount of Na^+^ reabsorbed by the CD can have a substantial impact on extracellular fluid volume and blood pressure (Mamenko et al. 2012).

Bradykinin (BK), the enzymatic product of kallikrein‐kinin system (KKS), is a major modulator of Ang II actions on blood volume, vascular reactivity and salt sensitivity (Carretero and Scicli 1995; Shen and El‐Dahr 2006; Shen et al. 2007). Angiotensin converting enzyme (ACE) is the corner stone of the balance between the levels of these two peptides (Brown and Vaughan 1998; Adam et al. 2001; Shen and El‐Dahr 2006). ACE inhibition by increasing BK further exerts anti‐hypertensive and cardio‐protective actions (Brunner et al. 1979; Braunwald 1991). The RAS and KKS cooperate at multiple levels, including: (1) The physical and functional interactions between AT_1_R and bradykinin type‐2 receptor (B_2_R), which potentiate the actions of the AT_1_R (AbdAlla et al. 2000, 2001); (2) The Ang II/AT_1_R‐mediated upregulation of *Bdkrb2* gene expression (Shen et al. 2007); (3) The co‐expression of kallikrein and renin in the distal nephron segments regulating the function of the CD (Rohrwasser et al. 2003); (4) Inhibition of ACE increases CD renin (Gonzalez‐Villalobos et al. 2011); and (5) B_2_R deficient mice have decreased renin mRNA and protein expressions in the kidney (Kang et al. 2008). Taken together, it is likely that coordinated actions of these two systems fine‐tune renal Na^+^ reabsorption. We hypothesize that the activation of B_2_R increases renin synthesis and release in the CD via PKC. To test this hypothesis, we used a mouse kidney cortical CD cell line (M‐1 cells) to primarily examine the intracellular signaling involved in the BK/B_2_R‐dependent regulation of renin in the CD. We demonstrated that BK increases renin synthesis and secretion via the activation of PKC and nitric oxide (NO) release.

## Material and Methods

### Treatments and antibodies

Bradykinin (B3259), H89 (PKA inhibitor, B1427), calphostin C (PKC inhibitor, C6303), L‐NAME (NOS inhibitor, N5751), Icatibant (HOE 140 B_2_R antagonist, H157) were purchased from Sigma‐Aldrich (Saint Louis, MO). For detection of prorenin and renin, we used a rabbit anti‐renin polyclonal IgG H‐105 antibody (sc‐22752), B_2_R was detected using anti‐B_2_R goat polyclonal IgG antibody (sc‐15050) and *β*‐actin was detected using a mouse anti‐*β*‐actin monoclonal IgG antibody (sc‐4778) all from Santa Cruz Biotechnology, Santa Cruz, CA. Anti‐rat aquaporin‐2 (AQP2) antibody was purchased from Abcam (Cambridge, UK). For Western blot experiments the secondary antibodies used were the IR Dye 800CW or 650 anti‐goat, mouse and rabbit according to the primary antibody chosen (Li‐Cor Bioscience, NE) and for immunofluorescence the secondary Alexa fluor antibodies (Alexa fluor‐488 or ‐594) were purchased from Life Technologies (Carlsbad, CA). M‐1 cell line was obtained from American Type Culture Collection (ATCC, CRL‐2038, Manassas, VA).

### Cultures of M‐1 cells

We used a mouse kidney cortical CD, SV40 transformed cells (M‐1 cells) that express many characteristics of the CD‐like epithelial morphology and CD‐specific antigens, exhibiting principal and intercalated cells functions (Stoos et al. 1991). M‐1 cells were cultured as previously described (Gonzalez et al. 2016), cell culture media was DMEN (ATCC 30‐2002), containing 0.48 mmol/L that is sufficient to induce NO production. Varying concentrations of BK ranged from 10^−14^ to 10^−6^ mol/L and vehicle (phosphate buffer saline, PBS, pH 7) was used as control. As indicated in the Figure Legends different pharmacological tools were added to dissect the molecular mechanism of BK and cells harvested after 6 h of incubation. For Western blot analysis, the cells were lysed in a buffer containing 1 mmol/L EDTA, 20 mmol/L HEPES‐Tris (pH 7.0), 250 mmol/L sucrose, and 0.15 mg/mL trypsin inhibitor, using a Potter‐Elvejhem homogenizer with a teflon pestle. Protein concentration determination by a bicinchoninic acid protein assay kit (Pierce, Rockford, IL). We used 5–6 different sets of cell cultures (control and experimental groups; *n* = 6) for each experimental design mentioned bellow.

### RNA isolation and quantitative real‐time RT‐PCR (qRT‐PCR)

For total RNA isolation, cells were washed with PBS and then total RNA was extracted using a commercially available kit (Qiagen, Hilden, Germany). RT‐PCR was performed to detect *Bdkr2b* in M‐1 cells as described in (Shen et al. 2007; Kang et al. 2008). The primers used were forward: 5′‐AGA‐ACC‐TCT‐TTG‐TCC‐TCA‐GCG‐3′ and reverse: 5′‐CGT‐CTG‐GAC‐CTC‐CTT‐GAA‐CT‐3′. To evaluate the renin gene expression qRT‐PCR was performed using the TaqMan PCR system as previously described (Gonzalez et al. 2011b; Lara et al. 2012b), and the data obtained were normalized to *β*‐actin mRNA expression levels. Primers used to amplify mRNA were: (1) renin (*Ren1C*) – forward: 5′‐AGT‐ACT‐ATG‐GTG‐AGA‐TCG‐GCA‐TT‐3′ and reverse: 5′‐AGA‐TTC‐ACA‐ACC‐TCT‐ATG‐ACT‐CCT‐C‐3′ and the probe 5′‐TTC‐AAA‐GTC‐ATC‐TTT‐GAC‐CAC‐GGG‐TTC‐AG‐3′ (2) *β*‐actin – forward: 5‐ATC‐ATG‐AAG‐TGT‐GAC‐GTT‐GA‐3′, reverse: 5′‐GAT‐CTT‐CAT‐GGT‐GCT‐AGG‐AGC‐3′ and probe: 5′/HEX/TCT‐ATG‐CCA‐ACA‐CAG‐TGC‐TGT‐CTG‐GT/BHQ2/3.

### Protein detection and quantification of B_2_R, renin, and its precursors

Thirty micrograms of total protein extract were separated by 10% SDS‐PAGE and transferred to a nitrocellulose membrane (Invitrogen, Carlsbad CA). For B_2_R protein detection in M‐1 cells, after blocking, the nitrocellulose membranes were incubated with a goat anti‐B_2_R polyclonal antibody for 1 h (1:400), followed by the incubation with a red fluorescent tagged anti‐goat secondary antibody (1:2000, IR Dye 650 donkey anti‐goat). For prorenin and renin proteins detection in M‐1 cells, we used a rabbit anti‐renin polyclonal antibody (1:1000), overnight 4°C, followed by the incubation of the membrane with a green fluorescent tagged anti‐rabbit secondary antibody (1:5000, IR Dye 800cw goat anti‐donkey). The primary antibody detected renin band at ~38 KDa and its precursors pre‐prorenin (~50 KDa) and prorenin (~48 KDa) corroborated elsewhere by using the recombinant mouse prorenin and renin as controls (Liu et al. 2011; Gonzalez et al. 2015). Moreover, the specificity of the antibody used was addressed by preadsorption of the renin antibody using 2× excess of purified recombinant human renin peptide (Liu et al. 2011). *β*‐actin protein expression was used as a loading control, after washing steps for removal of the anti‐renin antibody, the nitrocellulose membranes were blocked and incubated with primary mouse anti‐*β*‐actin monoclonal antibody for 45 min and a red fluorescent tagged anti‐mouse secondary antibody (1:5000, IR Dye 650 donkey anti‐mouse). Prorenin and renin quantifications were performed by densitometric analysis of the immunoreactive bands against *β*‐actin. Immunobands were detected by the Odyssey System (Li‐Cor Bioscience, NE) as described previously (Lara et al. 2012a).

### Renin and B_2_R immunofluorescence in M‐1 cells

M‐1 cells were incubated in an eight‐well chamber slides (Nunc Lab‐Tek Chamber Slide System, Sigma‐Aldrich, Saint Louis, MO). After executing the experimental groups (mentioned below), culture media were removed and the cells were fixed with 4% paraformaldehyde for 20 min, incubated with 0.1% Triton X‐100 for 3 min and blockade with Image‐iT FX signal enhancer (Invitrogen, Carlsbad, CA). For the co‐localization of renin and B_2_R in M‐1 cells, the cells were sequentially incubated with a rabbit anti‐renin polyclonal antibody (1:400, overnight at 4°C), its respective red secondary antibody (1:2000, Alexa fluor 594, for 45 min), followed by a goat anti‐B_2_R polyclonal antibody (1:200, for 1 h) and its respective green secondary antibody (1:1000, Alexa fluor 488). For evaluation of renin expression stimulated by BK, after addition (or not, control) of the peptide (10^−10^ mol/L) only with the rabbit anti‐renin polyclonal antibody and its respective red secondary antibody. ProLong Gold antifade reagent containing 4,6‐diamidino‐2‐phenylindole (Invitrogen, Carlsbad, CA) was used as a nuclear stain. Digital images, using a 100× oil‐immersion objective, were captured from 10 fields from three different set of cell culture using a digital DS‐U2/L2 USB camera attached to a Nikon Eclipse 50i fluorescence microscope.

### Quantification of renin content in the cell culture media

Renin content in cell culture media was determined by using modified protocols from PRA assay (GammaCoat Plasma Renin Activity ^125^I RIA kit [DiaSorin, Stillwater, MN]) as previously described (Gonzalez et al. 2015, 2016). Data were expressed by ng Ang I formed per hour per mL of cell culture media.

### cGMP levels

The cGMP levels of M‐1 cells were determined using a ELISA kit (cat #581021, Cayman, Ann Arbor, MI) according to the manufacture's instructions.

### Animal care and use of conventional and conditional knockout mice

The experimental animal protocols were approved by Tulane University Institutional Animal Care and Use Committees. Wild‐type mice and conventional knockout mice (BdkrB2^−/−^) (*n* = 4, each) were provided by Dr. Fred Hess and Dr. Howard Chen (Merck Research Laboratory, Rahway, NJ). Mice were generated on a C57BL/6J background and genotyped as previously described (Borkowski et al. 1995; El‐Dahr et al. 2000; Imig et al. 2003). The null mice had no visible phenotype, although mice have the phenotype of bradykinin insensitivity in several other tissues. Mice are fertile and indistinguishable from their littermates by visual inspection (Borkowski et al. 1995). The conditional knockout mice – which *Bdkrb2* gene was inactivated only in the CD (UB^*Bdkrb−*/−^ mice) – and control UB^*flox*/flox^ (*n* = 4, each) were generated by El‐Dahr laboratory at Tulane University Health Science Center, New Orleans, LA and the protocol was described elsewhere (Kopkan et al. 2015). At basal conditions, the conditional knockout presented the similar levels of SBP and urinary Na^+^ excretion (Kopkan et al. 2015). The immunofluorescence experiments were performed using the knockout mice at 10 weeks old.

### Immunofluorescence in renal tissue

To detect the immunoexpression of renin and B_2_R in the principal cells we used co‐localization with aquaporin 2 (AQP2), a marker of collecting duct principal cells, in paraffin embedded mice kidney sections (4 *μ*m) from wild‐type and knockout mice were processed by immunoperoxidase technique, as previously described (Lara et al. 2012a,b). Incubations of single sections and real consecutive sections were used for the co‐localization with: (1) rabbit anti‐renin polyclonal antibody (1:1000, overnight 4°C) and green anti‐rabbit secondary antibody (1:4000, Alexa fluor 488); (2) goat anti‐B_2_R polyclonal antibody (1:2000, 1 h) and red anti‐goat secondary antibody (1:5000, Alexa fluor 594); and (3) rat anti‐AQP2 polyclonal antibody (1:1000) and red anti‐rat secondary antibody (1:5000, Alexa fluor 594). Sections were blocked with horse serum before incubation with the primary antibody and between the consecutive steps of primary antibodies incubation. Digital images were captured from at least 15 fields of each four mice belonging from the controls, conventional and conditional knockout group with a digital DS‐U2/L2 USB camera attached to a Nikon Eclipse 50i fluorescence microscope.

### Statistical analysis

Data were expressed as mean ± SE. Statistical differences were accessed by one‐way ANOVA with Dunnet's post‐test. Significance was defined as *P* < 0.05.

## Results

### BK stimulates prorenin and renin in M‐1 cells

**Figure 1:**
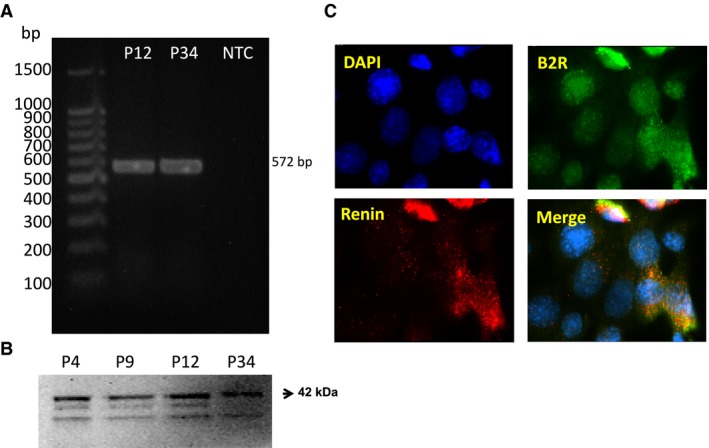
M‐1 cells express B_2_R. (A) Representative image of a PCR product detection of a specific single band of 572 bp, using primers for the *Bdkrb2*, in two different cell passages (P12 and P34) (*n* = 3). (B) Representative image of the immunodetection of a 42 KDa band comparable with the B_2_R molecular weight in different cell passages (P4, P9, P12, and P34) (*n* = 3). (C) Colocalization of B_2_R (green) and renin (red) in M‐1 cells. Image was captured using a 100× oil immersion objective and a DS‐U2/L2 USB digital camera attached to a Nikon Eclipse 50i fluorescence microscope.

We first examined the expression of B_2_R in M‐1 cells. The specific single band of the expected product (572 bp) obtained by PCR, as well as a 42 KDa band by immunoblotting, demonstrated the presence of the *Bdkrb2* gene and B_2_R in M‐1 (Fig. 1A and B). B_2_R and renin co‐localized in M‐1 cells (Fig. 1C).

**Figure 2:**
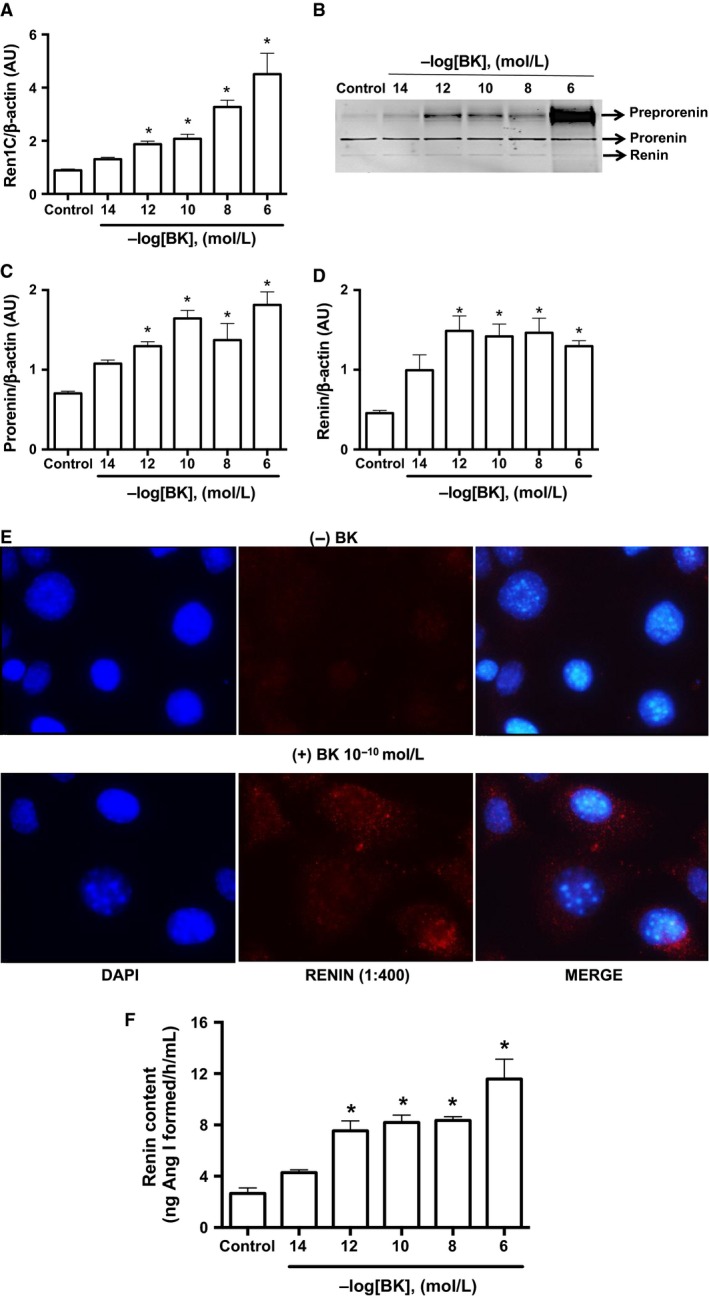
Bradykinin stimulates renin expression in M‐1 cells. (A) qRT‐PCR amplification of M‐1 cells renin (*Ren1C*) gene. M‐1 cells were incubated in the absence (control) or in the presence of different bradykinin concentrations (from 10^−14^ to 10^−6^ mol/L). Results were expressed as mean ± SE in arbitrary unities. (B) Representative image of renin protein detection by Western blot. Three bands were detected corresponding to pre‐prorenin (~48 kDa), prorenin (~45 kDa) and renin (~40 kDa). Densitometric analysis of either prorenin (C) or renin (D) bands was normalized against *β*‐actin densitometry. Results were expressed as mean ± SE in arbitrary units. (E) Immunofluorescence of renin (red) expression in M‐1 cells incubated without (top panels) or with bradykinin 10^−10^ mol/L (bottom panels) for 6 h. Representative images were obtained using a 100× oil immersion objective. (F) Renin content in the cell culture media after incubation with varying concentrations of bradykinin (from 10^−14^ to 10^−6^ mol/L). Results were expressed as mean ± SE in ng of Ang I formed/h/mL. In all graphs, significance (*) was defined as *P *<* *0.05 compared to control (*n *=* *5–6; one‐way ANOVA followed by Dunnet's post‐test).

Treatment of cultured M‐1 cells with increasing concentrations of BK (from 10^−14^ to 10^−6^ mol/L) for 6 h stimulated *Ren1C* gene expression in a dose‐dependent manner. At 10^−10^ mol/L, BK stimulated more than double the renin transcript compared to control (2.08 ± 0.17 AU vs. 0.89 ± 0.05 AU, *n* = 6, *P* = 0.0173) (Fig. 2A). Prorenin and renin protein contents, as well the immature form preprorenin, were also augmented by BK, (Fig. 2B). The maximal effect of BK on prorenin and renin protein contents was observed at 10^−12^ mol/L (prorenin: 1.30 ± 0.06 AU vs. 0.70 ± 0.03 AU, *n* = 6, *P* = 0.0007; renin: 1.49 ± 0.19 AU vs. 0.46 ± 0.03 AU, *n* = 6, *P* = 0.0039; Fig. 2C and D, respectively). Moreover, the specific renin immunoreactivity expressed as a punctuated pattern, increased in intensity in M‐1 cells treated with 10^−10^ mol/L BK for 6 h (Fig. 2E).

**Figure 3:**
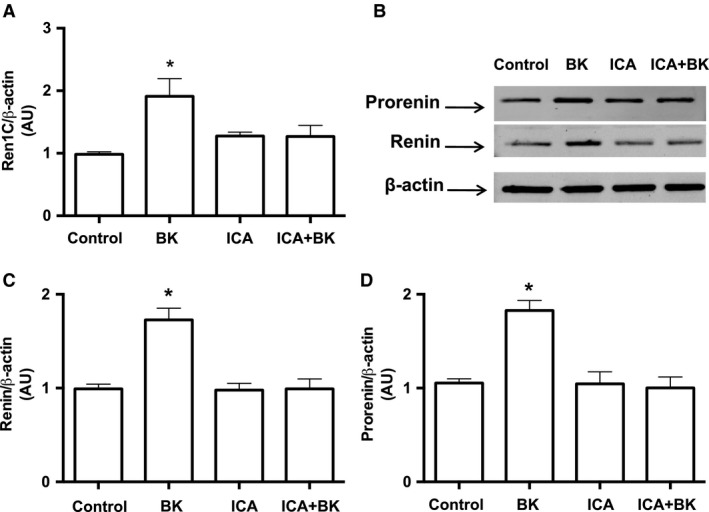
Bradykinin stimulates CD renin expression via B_2_R activation. (A) qRT‐PCR amplification of M‐1 cells renin (*Ren1C*) gene. Where indicated 10^−10^ mol/L bradykinin and 10^−6^ mol/L Icatibant (ICA), the B_2_R antagonist were added in the M‐1 cell culture media for 6 h. Results were expressed as mean ± SE in arbitrary unities. (B) Representative image of prorenin and renin detection by Western blot. Densitometric analysis of either prorenin (C) or renin (D) bands was normalized against *β*‐actin densitometry. Results were expressed as mean ± SE in arbitrary unities. In all graphs, significance (*) was defined as *P *<* *0.05 compared to control (*n *=* *5–6; one‐way ANOVA followed by Dunnet's post‐test).

To determine whether BK further increases renin release, we quantified renin content in the cell culture media. Increasing concentrations of BK stimulated renin in the extracellular media with a maximal effect at 10^−12^ mol/L. In this condition, renin content was threefold higher in treated cells as compared to control (7.54 ± 0.77 ng of Ang I formed/h/mL vs. 2.56 ± 0.43 ng of Ang I formed/h/mL, *n* = 6, *P* = 0.0106, Fig. 2F). BK‐dependent stimulations of *Ren1C* gene (Fig. 3A) and prorenin and renin contents (Fig. 3B–D) were mediated by B_2_R, because treatment the specific B_2_R antagonist Icatibant (10^−6^ mol/L) blunted these effects.

### Bradykinin stimulates renin in M‐1 cells via PKC and NO release, but not PKA

**Figure 4:**
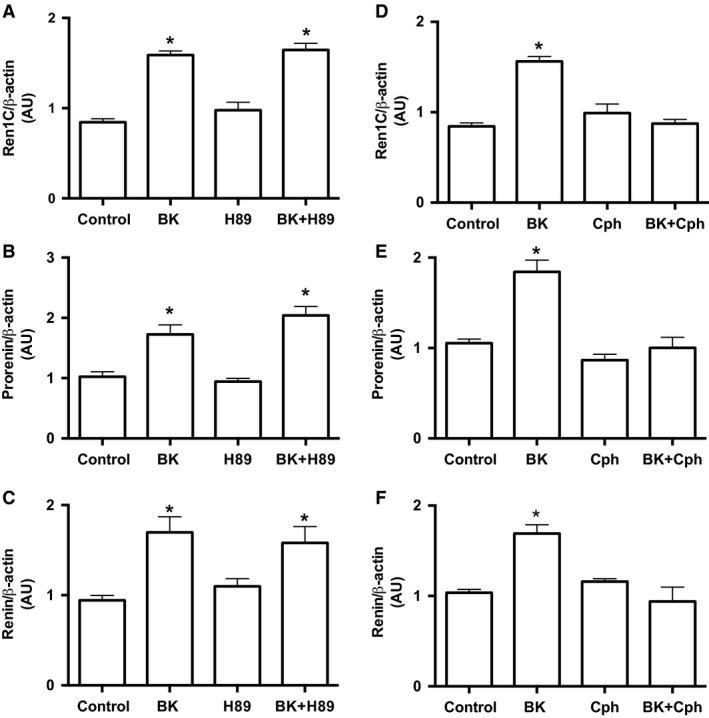
Bradykinin stimulates CD renin expression through a PKA‐independent and PKC‐dependent pathway. (A) and (D) qRT‐PCR amplification of M‐1 cells renin gene (Ren1C). Where indicated 10^−10^ mol/L bradykinin, 10^−7^ mol/L H89, a PKA inhibitor and 10^−7^ mol/L calphostin C, a PKC inhibitor were added in the M‐1 cell culture media for 6 h. Densitometric analysis of the specific bands (B) and (E) of either prorenin or (C) and (F) renin were normalized to *β*‐actin expression. Results were expressed as mean ± SE in arbitrary unities. In all graphs, significance (*) was defined as *P *<* *0.05 compared to control (*n *=* *5–6; one‐way ANOVA followed by Dunnet's post‐test).

To determine the intracellular pathway involved in the BK‐dependent stimulation of renin in M‐1 cells, we first treated M‐1 cells with BK in either the presence or absence of PKA inhibition with H89 (Fig. 4A–C). H89 (10^−7^ mol/L) did not alter the BK‐dependent stimulation of renin mRNA levels (Fig. 4A) and prorenin and renin contents (Fig. 4B and C). However, 10^−7^ mol/L calphostin C (Cph) – an inhibitor of DAG‐dependent PKC isoforms, completely abolished the stimulation of *Ren1C* gene (Fig. 4D) and prorenin and renin proteins (Fig. 4E and F) by BK.

**Figure 5:**
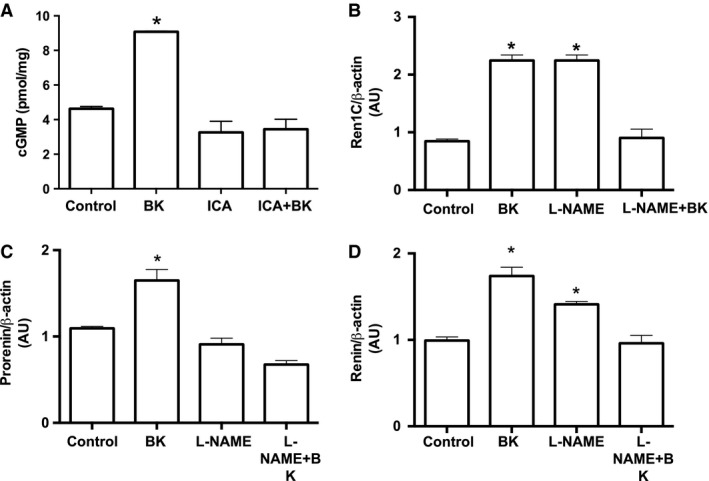
Bradykinin stimulates CD renin expression via NO release. (A) Measurement of cGMP levels (in pmol/mg of tissue) in the presence of 10^−10^ mol/L bradykinin and 10^−6^ mol/L Icatibant (ICA), the B_2_R antagonist were added in the M‐1 cell culture media for 6 h. Results were expressed as mean ± SE. (B) qRT‐PCR amplification of M‐1 cells renin gene (Ren1C). Where indicated 10^−10^ mol/L bradykinin, 10^−7^ mol/L L‐NAME, a NOS inhibitor were added in the M‐1 cell culture media for 6 h. Densitometric analysis of either prorenin (C) or renin (D) bands were normalized to *β*‐actin expression. Results were expressed as mean ± SE in arbitrary unities. In all graphs, significance (*) was defined as *P *<* *0.05 compared to control (*n *=* *5–6; one‐way ANOVA followed by Dunnet's post‐test).

Because B_2_R activation stimulates the NO/GMPc pathway, we further tested whether BK increases cGMP in M‐1 cells. Indeed, 10^−10^ mol/L BK augmented cGMP levels in M‐1 cells compared to controls (9.08 ± 0.04 vs. 4.63 ± 0.13 pmol/mg of protein, *n* = 3, *P* = 0.002), but his effect was prevented by treatment with 10^−6^ mol/L Icatibant (Fig. 5A). Figure 5B–D shows that the NO synthase inhibitor, L‐NAME (10^−6^ mol/L), abolished BK‐dependent stimulation of *Ren1C* gene and prorenin and renin proteins. Furthermore, L‐NAME, by itself increased *Ren1C* gene expression and renin and prorenin contents.

### Specific renin immunoexpression in the collecting ducts is decreased in mice with B_2_R deficiency

**Figure 6:**
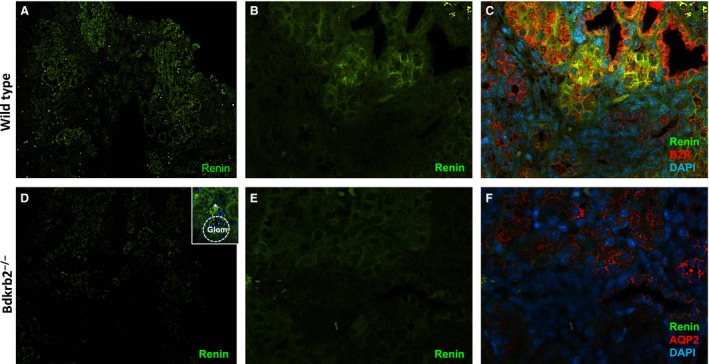
CD renin is decreased in mice lacking the *Bdkrb2* gene. Immunofluorescence was performed as described in Material and Methods section. Representative digital images from kidneys of wild‐type (A)–(B) and Bdkrb2^−/−^ (D)–(E) mice taken respectively at low (4× objective) and high (40× objective) magnifications show specific renin staining (green). (C) Co‐localization of renin (green) and B_2_R (red) in CD cells of kidneys from wild‐type mice. (F) Principal cells of the CD in Bdkrb2^−/−^ mice labeled with aquaporin‐2 (APQ‐2, red) showing the fainted renin (green) immunoexpression. Representative images were obtained using a 40× objective. The inset to Figure 1D denotes specific renin immunoexpression in JGA cells (*) in the Bdkrb2^−/−^ mice. Glomerulus (Glom) is highlighted by a dashed circle.

To assess if the *Bdkrb2* gene disruption alter the immunoexpression of renin in the CD, we used immunofluorescence in kidney sections from wild‐type, Bdkrb_2_^−/−^, control UB^*flox*/flox^ and UB^*Bdkrb2*−/−^ mice. Figure 6 shows microphotographs of kidney cortical sections from wild‐type and Bdkrb_2_^−/−^ mice at lower magnification (4× objective, Fig. 6A and B, respectively) and at higher magnification (40× objective, Fig. 6D and E, respectively) demonstrating reduced specific renin immunoexpression in the CD from the kidney of B_2_R null mice. As aforementioned in M‐1 cells, B_2_R and renin co‐localized in the CD of the wild‐type mice (Fig. 6C). However, in the kidneys from Bdkrb_2_^−/−^ mice, the AQP‐2 positive cells did not express specific renin staining (Fig. 6F). This finding was exclusively observed in CD cells since JGA cells showed renin immunoexpression (inset to Fig. 6D).

**Figure 7:**
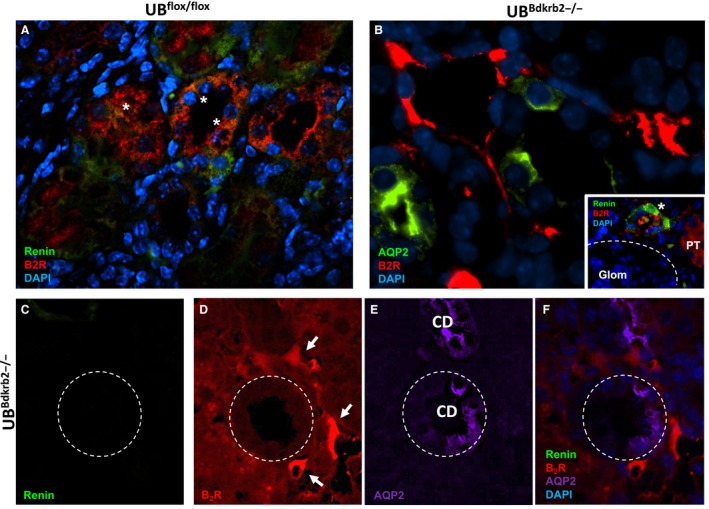
Specific renin immunofluorescence is decreased in the conditional UB^*Bdkrb2*−/−^ mice. (A) Co‐localization of renin (green) and B_2_R (red) in CD cells of a kidney section (3–4 *μ*m) from a control UB ^*flox*/flox^ mouse. (B) Localization of aquaporin 2 (AQP‐2, green) and B_2_R (red) in kidney CD cells in the conditional UB^*B*^ ^*dkrb2*−/−^. B_2_R immunoexpression was detected only in interstitial cells but not in the CD cells. (A)–(B) are representative images obtained using a 40× objective. The inset displays renin expression in JGA cells (asterisk) in the kidney of a Bdkrb2^−/−^ mouse. (C)–(F) Renin (green), B_2_R (red) and aquaporin‐2 (APQ‐2, violet) colocalization in the kidney CD cells in Bdkrb2^−/−^ mice. To obtain a 4‐color stain, we used two consecutive sections: in one section the antibodies against renin (green, C) and B_2_R (red, D) were incubated according to the Material and Methods section and in the second section renin (green, not showing) and AQP‐2 (red, E) antibodies were used. The red color of AQP‐2 detection was digitally changed by violet for better illustration (E). Renin immunoexpression was markedly reduced. The images obtained were merged (F). Representative images were obtained using a 100× objective. Arrows indicate expression of B_2_R in interstitial cells. CD is highlighted by dashed circles. Glomerulus is highlighted by a dashed circle. Glom, glomerulus; AA, afferent arteriole; PT, proximal tubule; CD, collecting duct.

The same pattern was observed in the CD from the kidneys of conditional B_2_R knockout mice. In control mice, B_2_R co‐localized with renin (Fig. 7A, indicated by the asteristic). As expected, in the conditional UB^*Bdkrb2*−/−^, B_2_R was detected in the interstitial cells but not in the CD cells identified by the AQP‐2 labeling (Fig. 7B). Yet, conditional UB^*Bdkrb2*−/−^ mice expressed renin in JGA cells (Inset to Fig. 7B). We used consecutive sections from conditional UB^*Bdkrb2*−/−^ mice to demonstrate the absence of specific renin immunostaining in AQP‐2 positive principal cells with negative B_2_R expression (Fig. 7C–F).

## Discussion

We previously demonstrated the synthesis and secretion of renin by the principal cells of the CD (Prieto‐Carrasquero et al. 2004; Gonzalez‐Villalobos et al. 2011). The present study provides evidence that the activation of B_2_R by BK increases renin synthesis and release in the CD cells via PKC and NO release in M‐1 cells, which support the notion, that BK/B_2_R activation represents a feed‐forward mechanism for renin in the CD.

Evidence of B_2_R expression in the renal CD has been previously reported by in vivo and in vitro studies. The expression of B_2_R in several segments of the nephron including distal tubules and CDs has been shown using autoradiography and electron microscopy in the rat kidney (Dean et al. 1997) and by immunohistochemistry and immunofluorescence in the kidney from BL57‐CJ mice (El‐Dahr et al. 2000; Kopkan et al. 2015). By in vitro evidence, B_2_R was also reported in inner medullary collecting duct (IMCD3) cells using immunohistochemistry and Western blot (Saifudeen et al. 2005) and pharmacological approaches in Madin‐Darbi canine kidney epithelial cell line (MDCK) (Slivka and Insel 1988). Our findings indicate that the activation of B_2_R increases prorenin and renin synthesis. Although we did not use cells with no expression of B_2_R as control, we performed immunofluorescence using two different mouse models, the null B_2_R knockout and the specific B_2_R knockout in the CD. In both models, the expression of CD renin was decreased.

The molecular mechanism involved in B_2_R‐stimulated prorenin and renin synthesis is dependent on PKC and independent of PKA activation. Previous work from our group demonstrated that the Ang II‐mediated stimulation of CD renin occurs through PKC activation because: (1) The phorbol ester PKC activator, PMA, mimicked the effects of Ang II in primary cultures inner medullary CD cells from Sprague‐Dawley rats (Gonzalez et al. 2011b); (2) The inhibition of PKC by calphostin C abolished the Ang II‐dependent stimulation of renin in M‐1 cells (Gonzalez et al. 2015); and (3) Transfection with PKC*α* dominant negative construct attenuated renin synthesis in response to Ang II in M‐1 cells (Gonzalez et al. 2015). Gomez et al. (2009) demonstrated that the activation of cAMP responsive element (CRE) of the *Ren1C* gene in JGA cells is required for renin phenotype maintenance. This activation is mediated by phosphorylation of the transcriptional factor CRE‐binding protein (CREB). Although, the main signaling pathway involved in CREB phosphorylation is cAMP/PKA, it has been demonstrated that PKC can also phosphorylates CREB (Brindle and Montminy 1992). Accordingly to previous studies (Gomez et al. 2009, 2014), our data suggest that the activation of CREB/CRE may be also the central molecular mechanism for renin synthesis in CD. Because CREB phosphorylation by PKC and PKA occurs at different consensus sites (ser‐121 or ser‐133, respectively) (Brindle and Montminy 1992), it is likely that the net phosphorylation of CREB drives *Ren1C* gene transcription (Gonzalez et al. 2015, 2016). There is growing evidence showing that marked differences in the regulation between JGA renin and CD renin are related to how Ang II triggers renin synthesis and release. In the secretion of renin by the JGA cells, cAMP is the dominant second messenger, while Ca^2+^ modulates the integrated activities of the enzymes related to cAMP synthesis and degradation (Churchill 1985; Schnermann and Briggs 2008; Atchison and Beierwaltes 2013). Moreover, direct increases in intracellular Ca^2+^ mobilization by using thapsigargin further inhibit *Ren1C* gene expression (Fray et al. 1987; Atchison and Beierwaltes 2013). As an atypical secretory phenotype, in JGA cells, Ca^2+^ does not directly affect JGA renin secretion (Grunberger et al. 2006; Ortiz‐Capisano et al. 2007). Ang II, which increases intracellular Ca^2+^ in the JGA cells, also inhibits cAMP and JGA renin (Kurtz and Wagner 1999). In contrast, in rodent models in which intratubular levels of Ang II are high (Von Thun et al. 1994; Prieto‐Carrasquero et al. 2004, 2008; Prieto et al. 2013), there is Ang II‐dependent stimulation of CD renin via AT_1_R and a Ca^2+^‐dependent PKC activation leading to cAMP accumulation and CREB phosphorylation (Gonzalez et al. 2011b, 2015). It is worth mentioning that in the present study, the involvement of cAMP/PKA pathway cannot be completely ruled out in vivo since activation of B_2_R increases prostaglandins, especially PGE_2_, which could further activate the cAMP/PKA pathway (Siragy et al. 1997; Steinert et al. 2009). Moreover, interstitial cells in the neighborhood of CD express cyclooxygenase‐2, the enzyme responsible for PGE_2_ production (Gonzalez et al. 2014a).

B_2_R‐mediated effect on CD renin also depends on cGMP. In the presence of L‐NAME, a NOS inhibitor, BK no longer increases renin transcript and renin and prorenin protein content. In JGA cells, it is known that NO exhibits a dual effect, either inhibits cAMP degradation, as a tonic enhancer of renin secretion (Kurtz and Wagner 1998; Castrop et al. 2004; Chaturvedi et al. 2007), or inhibits renin secretion through the activation of cGMP protein kinase (Kurtz and Wagner 1998). Our data suggest that in the CD cells, NO may act in coordination with PKC to release renin, because both L‐NAME and calphostin C completely abolished the BK/B_2_R‐dependent stimulation of renin. The fact that L‐NAME itself increases renin but not prorenin indicate that NO pathway might facilitate renin maturation. Furthermore studies are currently ongoing to address this issue.

Juxtaglomerular cells secrete mainly active renin (Castrop et al. 2010), while the principal cells of the CD primarily secrete prorenin (Kang et al. 2008; Prokai and Peti‐Peterdi 2010; Gonzalez et al. 2015). Our data indicate that B_2_R activation stimulates renin secretion because renin content increased in the cell culture media of M‐1 cells treated with physiological concentrations of BK (10^−12^ mol/L). According to a previous study (Campbell et al. 1993), the physiological level of BK in the kidney is about 100 fmol/g of wet kidney weight. Campbell et al. (1993), demonstrated that kidney levels of BK are much higher than circulating levels, suggesting local formation. Thus, it is possible that BK at a dose of 10^−12^ mol/L, the lowest concentration with response used in our study, be close to physiological levels.

Nevertheless, whether M‐1 cells primarily secrete either renin or prorenin or both in response to BK was not elucidated in the present study. Further studies are needed to examine the possibility that BK stimulates the secretion of prorenin in M‐1 cells that is subsequently cleaved in the extracellular space. Unfortunately, the in vitro observation of prorenin activation by kallikrein is not supported by in vivo studies using kallikrein knockout mice (Ramkumar et al. 2014). A potential mechanism to explain the activation of prorenin locally secreted in the distal nephron segments could be via the prorenin receptor (PRR) (Danser and Deinum 2005). The PRR is expressed on the apical membrane of intercalated cells in rats, mice, as well as in M‐1 cells (Gomez et al. 2009; Gonzalez and Prieto 2015a,b; Gonzalez et al. 2015, 2016). The contribution of the PRR to the non‐hydrolytic activation of prorenin and subsequent generation of intratubular Ang I and Ang II, is a subject of active investigation (Gonzalez et al. 2011a, 2014b; Huang and Siragy 2012).

Under physiological conditions, the KKS interacts with the RAS due to the dual function of ACE to degrade BK and stimulate Ang II production (Brown and Vaughan 1998; Adam et al. 2001; Shen and El‐Dahr 2006). We demonstrated, by immunofluorescence, in wild‐type mice the presence of B_2_R in the plasma membranes, with stronger labeling at the apical side of the collecting duct cells, but our study did not rule out the intracellular localization. Previous studies (Imig et al. 2003; Kopkan et al. 2015) using the null B_2_R knockout mice and mice with conditional deficiency of B_2_R in the CD (same knockout models of this study), demonstrated that *Ren1C* transcript and renin protein were diminished in whole kidney samples. Unfortunately, those studies did not examine the specific effects of B_2_R deficiency on either JGA renin or CD renin. The physiological and pathophysiological consequences of renin in the distal nephron segments have been investigated in CD‐specific renin knockout mice (Ramkumar et al. 2014). Ramkumar et al., showed that mean arterial pressure is attenuated in mice with renin deficiency in the CD chronically infused with Ang II, suggesting the involvement of the epithelial Na^+^ channel (ENaC). In this study, we show complementary evidence that null B_2_R knockout mice as well as mice with conditional deficiency of B_2_R in the CD, both exhibit reduced specific immunoreactivity in the CD. It is likely that the distal nephron segments possess a feed‐forward interaction between the KKS and RAS. In aortic vascular smooth muscle cells feed‐forward interaction of RAAS and KKS contributes to the vascular remodeling (Ceravolo et al. 2014) and the conditional B_2_R knockout mice, which do not express CD renin, exhibit an attenuated blood pressure response during Ang II‐dependent salt sensitive hypertension (Kopkan et al. 2015).

The presence of renin and B_2_R in AQP2‐positive principal cells along with the pharmacological effects tested by us indicate that at least in the principal cells, BK directly regulates renin via B_2_R activation. The potential paracrine regulation of renin by BK via B_2_R in the collecting duct cannot be discarded. We also detected B_2_R expression in the interstitial cells. It is likely that BK exerts a paracrine regulation of renin produced by neighboring principal cells in the CD. Interstitial cells in the renal inner medulla express cyclooxygenase‐2, the enzyme responsible for PGE2 production (Gonzalez et al. 2016). As mentioned above, B_2_R increases PGE_2_/cAMP/PKA pathway (Rohrwasser et al. 1999, 2003), acting as a paracrine stimulator of renin synthesis by the neighboring CD cells. Thus, in the present study, the conditional B_2_R knockout mice (UB^Bdkrb2−/−^) that do not express B_2_R in the CD and have marked suppression of renin expression in the principal cells, is an evidence that B_2_R in the CD drives the lead in the local regulation of renin as compared to B_2_R in the interstitial cells. Nonetheless, future studies using mice with conditional knockout of B_2_R in the CD are needed to examine the impact of B_2_R deficiency on CD renin and its functional consequences.

In summary, this study demonstrates that B_2_R activation regulates renin in the CD via two different mechanisms, via diacylglycerol‐dependent PKC signaling and CREB phosphorylation, as well as by NO release, which acts as a second messenger to activate protein kinase G resulting in renin synthesis and/or maturation. These findings concur with previous studies demonstrating that B_2_R deficient mice have decreased renin protein and mRNA expression and kidney Ang II levels (Imig et al. 2003). Collectively, our data support further the hypothesis that the B_2_R‐dependent regulation of renin in the collecting duct involves a feed‐forward mechanism, which may contribute to aggravate hypertension.

## Conflict of Interest

The authors declare no conflicts of interest.
